# A comparison of the prevalence of dry mouth and other symptoms using two different versions of the Edmonton Symptom Assessment System on an inpatient palliative care unit

**DOI:** 10.1186/s12904-024-01405-7

**Published:** 2024-03-16

**Authors:** Ragnhild Elisabeth Monsen, Anners Lerdal, Hilde Nordgarden, Caryl L. Gay, Bente Brokstad Herlofson

**Affiliations:** 1 Department of Interdisciplinary Health Sciences, Institute of Health and Society, Faculty of Medicine, University of Oslo, Postboks 1089 Blindern, Oslo, 0317 Norway; 2 Department of Medicine, Lovisenberg Diaconal Hospital, Oslo, Norway; 3 Department of Research, Lovisenberg Diaconal Hospital, Oslo, Norway; 4 National Resource Centre for Oral Health in Rare Disorders, Lovisenberg Diaconal Hospital, Oslo, Norway; 5 Department of Family Health Care Nursing, University of California, San Francisco, San Francisco, USA; 6 Department of Oral Surgery and Oral Medicine, Faculty of Dentistry, University of Oslo, Oslo, Norway; 7 Unit of Oral and Maxillofacial Surgery, Department of Otorhinolaryngology – Head and Neck Surgery Division for Head, Neck and Reconstructive Surgery, Oslo University Hospital, Oslo, Norway

**Keywords:** Advanced cancer, Symptom assessment, Edmonton Symptom Assessment System, Dry mouth, Open-ended question

## Abstract

**Background:**

Symptom assessment is key to effective symptom management and palliative care for patients with advanced cancer. Symptom prevalence and severity estimates vary widely, possibly dependent on the assessment tool used. Are symptoms specifically asked about or must the patients add them as additional symptoms? This study compared the prevalence and severity of patient-reported symptoms in two different versions of a multi-symptom assessment tool. In one version, three symptoms dry mouth, constipation, sleep problems were among those systematically assessed, while in the other, these symptoms had to be added as an “Other problem”.

**Methods:**

This retrospective cross-sectional study included adult patients with advanced cancer at an inpatient palliative care unit. Data were collected from two versions of the Edmonton Symptom Assessment System (ESAS): modified (ESAS-m) listed 11 symptoms and revised (ESAS-r) listed 9 and allowed patients to add one “Other problem”. Seven similar symptoms were listed in both versions.

**Results:**

In 2013, 184 patients completed ESAS-m, and in 2017, 156 completed ESAS-r. Prevalence and severity of symptoms listed in both versions did not differ. In ESAS-m, 83% reported dry mouth, 73% constipation, and 71% sleep problems, but on ESAS-r, these symptoms were reported by only 3%, 15% and < 1%, respectively. Although ESAS-r severity scores for these three symptoms were higher than on ESAS-m, differences did not reach statistical significance.

**Conclusion:**

We identified significant differences in patient symptom reporting based on whether symptoms like dry mouth, obstipation and sleep problems were specifically assessed or had to be added by patients as an “Other problem”.

**Supplementary Information:**

The online version contains supplementary material available at 10.1186/s12904-024-01405-7.

## Background

It is common for patients with advanced cancer to experience multiple symptoms during palliative care (PC). Enhanced symptom assessment and management is a primary goal in cancer and PC and is associated with improved patient quality of life [1, 2]. Symptom frequency and severity, as well as how patients experience and report symptoms, varies according to diagnosis, cancer stage, treatment-related effects and comorbid conditions, but also by the assessment tool used [3]. In a study investigating symptom prevalence among 1507 patients receiving PC for cancer, lack of energy, pain, drowsiness, and dry mouth were the four most common symptoms [4]. Accurate information about symptom prevalence and severity is important for both clinical practice and research on symptom burden and management [5, 6], and monitoring over time may increase the health care professional’s awareness of symptoms that may fluctuate over time due to changes in the patient’s condition [7].

To identify bothersome conditions, a systematic approach with patient-reported outcome measures (PROMs) forms the basis for effective symptom assessment, monitoring and management [8–10]. Several validated questionnaires exist for assessing symptoms and quality of life in palliative care, both for research and in clinical practice [11, 12]. Some questionnaires include only a short list of symptoms to reduce the burden on critically ill patients [13–16]. However, patients with advanced cancer tend to have an average of eleven to thirteen symptoms [17–19], and the criticism of shorter tools has been that they may fail to capture a comprehensive and individualized profile of patients’ total symptom burden [20, 21].

To balance the need for assessment tools to be both short and comprehensive, some include the option for patients to add other relevant symptoms and problems in addition to those listed [16, 22–24]. Symptoms identified in such “open” questions are rarely reported in research [25–27]. A previous study identified dry mouth as a prioritized symptom among patients with advanced cancer [28]. However, because dry mouth was not included in the study’s validated questionnaires, the symptom was excluded from subsequent analysis. Assessing only selected symptoms in this way may result in a skewed picture of the patterns of symptom burden in the palliative care population.

It has been shown that estimates of symptom prevalence and severity are highly dependent on whether the symptom is systematically assessed (i.e., specifically asked about) or needs to be self-reported by patients, often by adding it as an “other symptom” [19, 29–31]. These studies estimated symptom prevalence using different assessment methods and therefore their results may not be directly comparable. Nonetheless, patients were less likely to report symptoms that were not systematically assessed as part of a symptom list. In addition, it has been shown that only moderate to severe symptoms tend to be reported when patients have to write them in [27]. As a result, relying on patients to report symptoms not listed in standard assessment tools, may lead to under-reporting and identification of symptoms and prevent or delay management and treatment of bothersome patient problems [32]. Although these prior research findings indicate that assessment methods influence patients’ reporting of symptoms, these studies compared different methods, such as listed symptoms versus interview or symptoms described in medical records [19, 20, 30].

Thus, the primary aim of this study was to compare the prevalence and severity of the symptom dry mouth in patients receiving palliative care for advanced cancer using two different versions of the Edmonton Symptom Assessment System (ESAS), is the most used and recommended tool in Norwegian palliative care settings. One ESAS version systematically assessed symptoms as part of a longer symptom list, while the other omitted some symptoms from the list, but allowed patients to add them as an “Other problem”. Furthermore, we investigated the prevalence of constipation and sleep problems, aiming to determine whether these symptoms exhibited a comparable pattern to dry mouth in terms of being reported under the category of “Other problem”.

Based on prior findings, we hypothesized that symptom prevalence would be higher when a symptom was included in the symptom list compared to when it had to be added by the patient. In addition, we hypothesized that symptoms that had to be added by the patient would be rated as more severe compared to when the same symptom was included in a symptom list.

## Methods

### Study design and samples

This retrospective cross-sectional study used data from two different versions of the Edmonton Symptom Assessment System (ESAS) collected from medical records at Lovisenberg Palliative Care Centre (LPCC), formerly Hospice Lovisenberg, at Lovisenberg Diaconal Hospital (LDS) in Oslo, Norway. This study is part of the Oral Health in Advanced Cancer (OralHAC) project [33] with the overall aim to investigate aspects of oral health in patient care in late-stage cancer by addressing and managing oral symptoms and discomfort. The project was approved by the Regional Committee for Medical Research Ethics, Health Region South-East Norway (reference #2013/1531).

The LPCC inpatient unit offers palliative care and treatment for patients with incurable cancer or other serious chronic illness and for dying patients. Patients who qualify for admission to the unit are initially offered a 14-day stay, although if the patient is dying, their stay will be extended. If the patient is in a terminal stage, with life expectancy of weeks and month, they will be transferred for community care at home or in a nursing home. In 2013, the 291 patients admitted to the inpatient unit had an average stay of 13 days, and 143 patients (49%) died in the unit. In 2017, the 321 patients admitted had an average stay of 12 days, and 148 (46%) died in the unit.

PC providers in the unit include physicians and nurses, and a physiotherapist, social worker, dietitian, occupational therapist, and priest. The team promotes a holistic approach and support for the patient and their relatives, and symptom monitoring and management are primary targets. All patients at the unit are above 18 years of age, and the majority are diagnosed with incurable metastatic cancer. All those who are able complete the ESAS questionnaire when they enter the unit, by themselves or with assistance from the nurses in the unit. Their responses are evaluated during the patient’s intake interview and by the physician and nurses for further care planning.

Patients admitted to the inpatient LPCC in 2013 or 2017 were identified for study inclusion. Patients eligible for the study had incurable metastatic cancer and were admitted to the inpatient palliative care unit between January and December 2013 or between January and December 2017. Patients missing a baseline ESAS or who had previously been admitted to the unit were excluded.

### Symptom measures

The outcomes of this study were patient-reported symptom prevalence and intensity, as measured with either of two different versions of the Norwegian ESAS questionnaire. In 2013, a modified version of the ESAS (ESAS-m) was used, and in 2017, a revised version (ESAS-r) was implemented.

### ESAS

The original ESAS was a self-report symptom assessment tool published in 1991 [15]. ESAS was designed to determine the presence and severity of eight common symptoms (i.e., pain, tiredness, nausea, depression, anxiety, drowsiness, appetite, wellbeing), with an option for patients to add one additional patient-specific symptom. Over the years, the tool evolved from a visual assessment scale (VAS) to an 11-point numeric rating scale (NRS), ranging from 0 (symptom is absent) to 10 (worst possible severity), and a ninth symptom, shortness of breath, was added [34].

The recommended cut-off for clinically relevant symptom burden is ≥ 4 on the 0–10 NRS [35]. In addition, ESAS symptom severity scores have been categorized as: none (0), mild [1–3], moderate [4–6], and severe [7–10, 34]. There is currently no consensus about the Minimal Clinically Important Difference (MCID), or the smallest magnitude of change that is clinically meaningful, for the ESAS’ individual symptom scores. Some studies report a universal MCID of one point in either direction for all symptoms [34, 36], while others report higher MCID values or different MCID values for improvement and deterioration or across different ESAS items [37–39].

### Modified ESAS (ESAS-m)

The original ESAS [15] was translated into Norwegian in 1999 [40]. During the translation process, the symptoms “dry mouth”, meaning the subjective sensation of dry mouth, and “pain on movement” were added, and “drowsiness” and the option to add a patient-specific “Other problem” were omitted [40]. This translated version was slightly modified into a local version (ESAS-m) and implemented at LDS in 2005, with the addition of two extra symptoms, “constipation” and “sleep problems” (see Supplementary File 1). This locally-modified version assessed overall wellbeing and 11 symptoms: pain at rest, pain on movement, tiredness, nausea, shortness of breath, dry mouth, lack of appetite, constipation, sleeping problem, anxiety and depression. The ESAS-m used at LDS assessed symptoms occurring “in the past day”.

### Revised ESAS (ESAS-r)

The ESAS-r is a revised version of the original ESAS [15]. The ESAS-r was validated and published in 2011 [24]. The list of symptoms was revised, brief symptom descriptions were added, and the timeframe was changed from “in the last 24 hours” to “now” [41]. This new version was translated into a number of languages, including Norwegian in 2012 [40], and implemented at LPCC in 2014. The translated ESAS-r asks about overall wellbeing and nine symptoms: pain, fatigue, drowsiness, nausea, lack of appetite, shortness of breath, depression and anxiety. The ESAS-r also allows patients to add one “Other problem, for example constipation” and rate its severity on the same NRS. However, the symptoms dry mouth, constipation and sleep problems were not included in the translated ESAS-r version, but could be reported as an “Other problem”, suggested with the symptom constipation.

### Demographic and clinical characteristics

Data on patient age (years), sex (male vs. female), cohabitation status (married/cohabitant vs. not), cancer diagnosis according to the International Classification of Disease (ICD)-10 system (grouped as shown in Table 1), metastasis (no vs. yes), and the number and type of prescribed medications, were collected from patients’ medical records.

### Statistical analysis

Data were analyzed using SPSS version 28.0 for Windows (IBM Corp, Armonk, NY). Descriptive statistics were used to summarize demographic, clinical, and symptom data. Continuous variables were presented as means and standard deviation (SD), and categorical variables as frequencies and percentages. Baseline characteristics were compared using independent sample t-tests for continuous variables and chi-square tests for categorical variables.

The prevalence of each symptom was calculated as the proportion of severity scores greater than 0 on the 0–10 NRS. A severity score of 0 indicated the symptom was absent. For the symptoms of constipation, dry mouth, and sleep problems, which were not systematically assessed in the ESAS-r, a severity score of 0 was assigned if the symptom was not added by the patient as an “Other problem” (this was done for the ESAS-r sample only, as these symptoms were systematically assessed in ESAS-m). For 2017 patients who added these symptoms in the ESAS-r as an “Other problem”, the symptom was considered present if the patient’s severity rating for that symptom was > 0. Chi-square tests were used to compare the prevalence of each symptom in the ESAS-m and ESAS-r samples.

To avoid confounding symptom prevalence and severity, comparisons of symptom severity scores were restricted to patients reporting each symptom (i.e., rating its severity > 0). Patients for whom a symptom was absent (i.e., rated its severity as 0) were excluded from the comparison of that symptom’s severity. Independent sample t-tests were used to compare symptom severity scores on the ESAS-m and ESAS-r. Because the pain at rest and pain on movement items in ESAS-m were replaced by a single pain item in ESAS-r, severity scores on the single pain item were compared to both ESAS-r pain items.

All comparisons were two-sided, using a 5% level of significance (CI 95%). Cohen’s d values were calculated to estimate the effect size of the group differences, with 0.20 considered small, 0.50 medium and 0.80 large [42].

## Results

### Sample characteristics

A total of 612 patients were admitted to the palliative unit in 2013 and 2017. Of 491 eligible patients for the study, 340 had baseline ESAS-m (2013) or ESAS-r (2017) data included in their medical records. Of these, 184 were recruited from the year 2013 and 156 from 2017. Sample characteristics for both groups are summarized in Table 1. There were no statistically significant differences in demographics or clinical characteristics between the two patient groups.

**Table 1:** Demographic and clinical characteristics of the ESAS-m^a^ (2013) and ESAS-r ^b^ (2017) patient samples

Characteristic	ESAS-m^a^ (*n* = 184)	ESAS-r ^b^ (*n* = 156)	*P* = value
Age, years			
Mean (SD)	64.6 (12.7)	66.7 (10.7)	*p =* .10
Range	29–93	32–91	
Sex, % (n)			*p* > .99
Male	43(76)	42 (65)	
Female	59 (108)	58 (91)	
Cohabitation status, % (n)			*p =* .80
Married/cohabitant	41 (76)	57 (88)	
Primary diagnosis % (n)			*p =* .48
Gastrointestinal cancer	23 (41)	30 (47)	
Lung cancer	23 (41)	17 (26)	
Breast cancer	13 (23)	11 (17)	
Gynecologic cancer	10 (19)	10 (15)	
Genital cancer male	8 (15)	7 (11)	
Brain Cancer	3 (6)	1 (2)	
Other cancer	21 (39)	24 (38)	
Metastases			*p =* .78
Yes, % (n)	84 (155)	86 (134)	
Number of prescribed medications			*p =* .48
Mean (SD)	8.5 (3.6)	8.2 (3.2)	
Range	1–19	2–17	
Type of medical treatment, % (n)			
Opioids	83 (153)	78 (122)	*p =* .31
Non-opioids pain medication	57 (105)	51 (79)	*p =* .28
Corticosteroids	61 (112)	49 (77)	*p =* .43
Anti-depressants	25 (46)	21 (33)	*p =* .48
Benzodiazepines	47 (85)	37 (58)	*p =* .12

^a^ ESAS-m is a 12-item modified version of the original Edmonton Symptom Assessment Scale (ESAS) that was in use at LPCC in 2013.

^b^ ESAS-r is the revised version of the original ESAS that was in use at LPCC in 2017.

### Comparisons of symptom prevalence

**Fig. 1:**
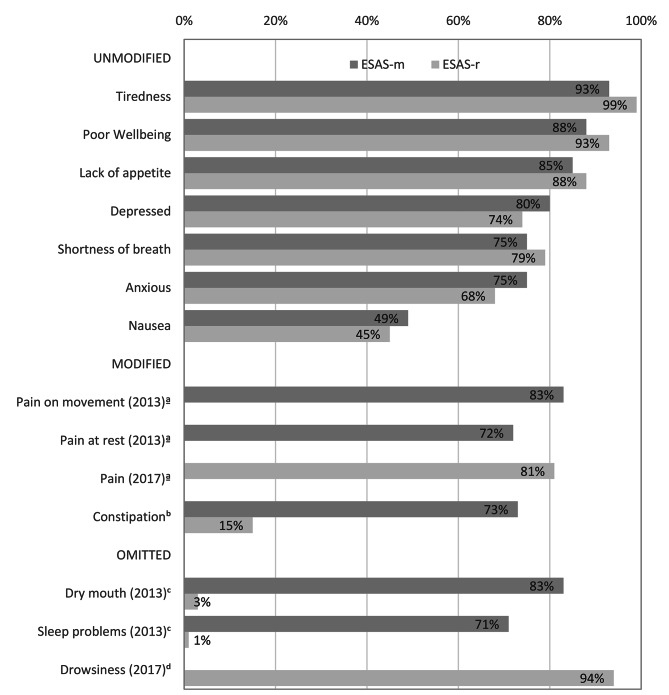
Comparison of symptom prevalence (severity ratings > 0) for two versions of the Edmonton Symptom Assessment Scale (ESAS): the 12-item modified version (ESAS-m) in use in 2013 and the 10-item revised version (ESAS-r) in use in 2017. ^a^ The pain at rest and pain on movement items in the ESAS-m were replaced by a single pain item in the ESAS-r. ^b^ Constipation was not systematically assessed in the ESAS-r, but was given as an example of an “Other problem” and some patients reported it. ^c^ Dry mouth and sleep problems were not mentioned in the ESAS-r, but a few patients reported them as an “Other problem”. ^d^ Drowsiness was omitted from the ESAS-m, but was included in the ESAS-r. Because the ESAS-m did not allow for additional symptoms to be written in, its prevalence using the ESAS-m could not be determined

Table 2 summarizes the prevalence of each symptom, as determined by ESAS-m and ESAS-r. For the seven symptoms assessed similarly in both versions, the only significant differences in symptom prevalence were that tiredness was more prevalent in ESAS-r than in ESAS-m (*p* = .02), and pain at rest (*p* = .04) in ESAS-m was more prevalent than pain (in general) in ESAS-r. In contrast, the three symptoms dry mouth, constipation and sleep problems were highly prevalent in ESAS-m, but were reported at dramatically lower rates in ESAS-r where patients had to self-report these symptoms as an “Other problem” (Fig. 1). Constipation was reported by 73% of patients in ESAS-m, but even though it was suggested as an example in “Other problem” in ESAS-r, it was only reported by 15% of patients. Even more striking was the 83% prevalence of dry mouth in ESAS-m, compared to a 3% prevalence in ESAS-r, and the 71% prevalence of sleep problems in ESAS-m compared to < 1% prevalence in ESAS-r. The prevalence of drowsiness could not be compared for the two versions because ESAS-m did not include it in the symptom list and did not include the option to write it in.

**Table 2:** Comparison of symptom prevalence (defined as the proportion of severity ratings > 0) using ESAS-m^a^ in 2013 versus ESAS-r^b^ in 2017

Symptoms	Symptom prevalence, % (n/N)			
	ESAS-m^a^ (*N* = 184)	ESAS-r^b^ (*N* = 156)		*p*-value
*Items listed similarly in both versions*				
Tiredness	93 (170/182)	99 (151/153)		*p* = .02
Nausea	49 (90/182)	45 (69/154)		*p* = .40
Lack of appetite	85 (153/180)	88 (135/153)		*p* = .39
Shortness of breath	75 (136/181)	79 (21/154)		*p* = .46
Anxious	75 (136/182)	68 (100/147)		*p* = .18
Depressed	80 (141/177)	74 (115/151)		*p* = .45
Wellbeing	88 (152/173)	93 (125/135)		*p* = .17
*Items mentioned in both versions but in different ways*				
Pain at rest^c^	72 (131/182)			*p* = .04
Pain on movement^c^	83 (149/180)			*p* = .74
Pain^c^		81 (127/156)		
Constipation^d^	73 (129/176)	15 (24/156)^b^		*p* < .001
*Items listed in only one version*				
Dry mouth^e^	83 (151/183)	3 (5/156)^c^		*p* < .001
Sleep problems^e^	71 (126/178)	< 1 (1/156)^c^		*p* < .001
Drowsiness^f^		95 (147/155)		n/a

^a^ ESAS-m is a modified version of the original Edmonton Symptom Assessment Scale (ESAS) in use at the palliative care unit in 2013 and included 12 items as described in this table. ^b^ ESAS-r is the revised version of the original ESAS in use at the palliative care unit in 2017 and included 10 items as described in this table. ^c^ The pain at rest and pain on movement items in the ESAS-m were replaced by a single pain item in the ESAS-r. Each of the ESAS-m pain items was compared to the ESAS-r pain item. ^d^ Constipation was not systematically assessed in the ESAS-r, but was given as an example of an “Other problem” and some patients reported it. ^e^ Dry mouth and sleep problems were not mentioned in the ESAS-r, but a few patients reported them as an “Other problem”. ^f^ Drowsiness was omitted from the ESAS-m, but was included in the ESAS-r. Because the ESAS-m did not allow for additional symptoms to be written in, its prevalence using the ESAS-m could not be determined.

### Comparisons of symptom severity

The mean symptom severity scores for the two ESAS versions are summarized in Table 3. For the seven symptoms similarly listed in both versions, no significant differences in symptom severity scores were found. Among patients reporting pain in general on ESAS-r, the severity score was significantly lower than the severity score among patients reporting pain on movement in the ESAS-m, but did not differ for pain at rest. The numbers of patients who added dry mouth (*n* = 5), constipation (*n* = 24) or sleep problems (*n* = 1) in ESAS-r were small, which severely limited the statistical power to detect differences in severity scores. Although, none of the differences reached statistical significance, the severity scores for these three symptoms were all in the hypothesized direction, with higher severity scores for the few patients who added these symptoms in ESAS-r compared to those who had these symptoms listed in ESAS-m. For dry mouth, the effect size was in the moderate-large range, and for constipation (which was given as an example in ESAS-r), the effect size was in the small-moderate range. Only one patient added sleep problems as an extra symptom in ESAS-r, which limited the reliability of the estimated severity ratings.

**Table 3:** Comparisons of symptom intensity ratings on ESAS-m^a^ (used in 2013) and ESAS-r^b^ (used in 2017) among patients reporting each symptom as present (NRS > 0)

Symptoms	Symptom Severity Rating, mean (SD) [n]		*p*-value (95% CI of the group difference)	Cohen’s d (95% CI)
	ESAS-m^a^ (*n* = 184)	ESAS-r^b^ (*n* = 156)		
*Items in both versions*				
Tiredness	5.9 (2.3) [170]	5.9 (2.2) [151]	*p* = .93 (-0.47, 0.52)	*d* = 0.01 (-0.21, 0.23)
Nausea	3.4 (2.4) [90]	3.3 (2.2) [69]	*p* = .87 (− 0.67, 0.79)	*d* = 0.03 (-0.29, 0.34)
Lack of appetite	5.4 (2.7) [153]	5.9 (2.8) [135]	*p* = .12 (-1.14, 0.13)	*d*=-0.18 (-0.42, 0.05)
Shortness of breath	4.6 (2.7) [136]	4.5 (2.3) [121]	*p* = .55 (− 0.43, 0.80)	*d* = 0.07 (-0.17, 0.32)
Anxious	4.2 (2.4) [136]	4.1 (2.6) [100]	*p* = .72 (-0.52, 0.76)	*d* = 0.05 (-0.21, 0.31)
Depressed	4.3 (2.5) [141]	4.0 (2.4) [115]	*p* = .41 (-0.35, 0.86)	*d* = 0.10 (-0.14, 0.35)
Wellbeing	4.6 (2.2) [152]	4.8 (2.3) [125]	*p* = .45 (-0.73, 0.32)	*d*=-0.09 (-0.33, 0.15)
*Items mentioned in both versions but in different ways*				
Pain at rest ^c^	3.4 (2.1) [131]		*p* = .11 (-0.94, 0.12)	*d*=-0.20 (-0.44, 0.05)
Pain on movement ^c^	4.5 (2.2) [149]		*p* = .02 (0.13, 1.23)	*d* = 0.29 (0.05, 0.53)
Pain ^c^		3.8 (2.2) [127]		
Constipation ^d^	4.8 (2.7) [129]	5.8 (2.4) [24]	*p* = .11 (-2.10, 0.21)	*d*=-0.36 (-0.80, 0.08)
*Items in only one version*				
Dry mouth ^d^	5.2 (2.6) [151]	7.2 (2.0) [5]	*p* = .09 (-4.38, 0.35)	*d*=-0.77 (-1.66, 0.13)
Sleep problems ^d^	4.5 (2.4) [126]	5.0 (-) [1]	*p* = .82 (-5.24, 4.26)	*d*=-0.21 (-2.17, 1.76)
Drowsiness ^f^		5.3 (2.3) [147]	n/a	n/a

Note: ESAS, Edmonton Symptom Assessment Scale; NRS, Numeric Rating Scale (range 0–10); CI, confidence interval

^a^ ESAS-m is a modified version of the original ESAS in use at the palliative care unit in 2013 and included 12 items as described in this table

^b^ ESAS-r is the revised version of the original ESAS in use at the palliative care unit in 2017 and included 10 items as described in this table

^c^ The pain at rest and pain on movement items in the ESAS-m were replaced by a single pain item in the ESAS-r. Each of the ESAS-m pain items was compared to the ESAS-r pain item

^d^ Constipation was not systematically assessed in the ESAS-r, but was given as an example of an “Other problem” and some patients reported it

^e^ Dry mouth and sleep problems were not mentioned in the ESAS-r, but a few patients reported them as an “Other problem”

^f^ Drowsiness was omitted from the ESAS-m, but was included in the ESAS-r. Because the ESAS-m did not allow for additional symptoms to be written in, its severity using the ESAS-m could not be determined

## Discussion

The present study investigated the prevalence and intensity of common symptoms among patients receiving inpatient PC for advanced cancer, using different methods of multi-symptom assessment. Our findings showed substantially lower reporting of the three symptoms dry mouth, constipation and sleep problems when these symptoms were not listed in the assessment tool, but rather had to be written in as an “Other problem”. For all three symptoms, treatment and palliation is available to reduce discomfort. As a result, inadequate identification of these and other symptoms of importance to patients may prevent them from receiving optimal symptom control.

### Prevalence

Dry mouth was severely under-reported or nearly absent when patients had to write the symptom in on the ESAS-r (Fig. 1). Even though constipation was provided as an example of an “Other problem” (thereby drawing attention to it and alleviating the burden of recalling it as a symptom), only 15% reported it compared to 73% when it was listed among the symptoms assessed. The difference was even larger for dry mouth, which was the third most prevalent symptom in ESAS-m (83%), but only five patients (3%) added it as an additional symptom in ESAS-r. It is also worth noting that dry mouth had a higher prevalence than constipation in ESAS-m, but the opposite occurred using ESAS-r, suggesting that the versions do not even provide consistent estimates of relative prevalence (i.e., which symptoms are more or less prevalent than others). It is also important to note that ESAS-r only allows patients to report one other additional symptom. Thus, if patients experience more than one other symptom, they will have to choose which one to report, and based on these results, it seems that patients are most likely to report the example provided, in this case constipation [26].

A pertinent question would be whether dry mouth, constipation and sleep problems were relevant symptoms for the patients in our study, despite their decreased prevalence in 2017. Common symptoms reported in PC are pain, fatigue, weakness, anorexia, lack of energy, dry mouth, constipation, early satiety and dyspnea [17, 18]. Dry mouth and constipation, when assessed, are endorsed to be among the ten most-frequently reported symptoms among patients receiving PC [29, 30]. In cancer care, dry mouth, constipation and sleep problems have been associated with considerable discomfort and decreased quality of life [43–51]. In addition, dry mouth has been identified as the third or fourth most common symptom overall in patients with advanced cancer [30, 52–54]. Despite this knowledge, only constipation and sleep problems are most often integrated in multi assessment symptom tools, and in modified versions of ESAS [13, 16, 55–57], while dry mouth has not been added to the same extent, appearing in only a few generic symptom assessment tools [12, 32, 58, 59] and hardly any modified versions of ESAS [60].

Symptom prevalence studies relying on data obtained from cancer registry databases, using short instruments such as ESAS, may present a skewed picture of patients’ symptom burden [61–63]. They typically identify fatigue, drowsiness, and lack of appetite as most prevalent, which corresponds with our results using ESAS-r. However, when using ESAS-m, tiredness, lack of appetite and dry mouth were the three most frequently reported symptoms. Although ESAS-r allows additional bothersome symptoms to be reported as an “Other problem”, such symptoms are rarely reported in studies [26, 28], and therefore infrequently included in overall symptom analysis.

### Severity

Patients rarely added dry mouth, constipation or sleep problems as an “Other problem” in ESAS-r, but the mean severity scores for these symptoms were higher than when they were systematically assessed in ESAS-m. Although none of the differences were statistically significant, likely due to the small numbers of patients adding these symptoms on ESAS-r, the effect size for dry mouth was moderate to large and exceeded the 1-point MCID for the ESAS. Moreover, in contrast to other symptoms and the ESAS-m, the mean severity score for dry mouth using the ESAS-r was in the severe range, suggesting that more mild experiences of this symptom rarely were reported as an “Other problem”. The effect sizes for constipation and sleep problems were small to moderate, but the difference in constipation severity scores equaled the MCID. Only one patient added “sleep problems” in ESAS-r, limiting the usefulness of this analysis.

High severity scores for symptoms written in as an “Other symptom” is in line with other studies. In Rojas-Concha’s study (2020), patients were able to add three other symptoms/problems to the EORTC QLQ-C15-PAL, and 85% of the symptoms added in these open-ended questionnaires were rated as being of moderate to severe intensity [27]. In another study, among symptoms that had to be added by patients, 32% were rated as moderate and 51% as severe, with severe symptoms being 4.3 times more likely to be added than mild symptoms [30]. Patients’ under-reporting of mild symptoms may prevent clinicians from early identification and management of symptoms, potentially leading to negative impact on survival, quality of life and health care use [2].

### Clinical implications

Systematic assessment and management of bothersome symptoms are highly recommended. Although there is currently no ideal assessment tool with respect to minimizing patient burden and maximizing comprehensiveness [64], use of validated questionnaires for both research and clinical practice is recommended [12]. The ESAS-r questionnaire is recommended and extensively employed, and it is therefore critical to careful be aware of the selection of the symptoms included in this assessment tool. However, it is worth noting that recent modifications of the ESAS-r questionnaire [55, 57, 60] cover only a few symptoms. Dry mouth is often neglected in the inclusion of symptoms, despite its potential occurrence in conjunction with other oral symptoms in a clustered manner and decreasing quality of life [65].

Effective symptom control is a major focus of PC, and the limitations of ESAS-r prevent the assessment of symptom clusters. A longitudinal prospective study investigating symptom clusters in patients with advanced cancer added 10 symptoms to the ESAS-r to address this issue [66]. The study reported dry mouth (82.7%) as the third most prevalent symptom, and it occurred in a cluster with sleep impairment and anxiety, and in cluster with pain, weight loss and lack of memory [66]. In addition, the study conclusion highlighted the importance of regular assessment of symptoms and symptom clusters due to their prognostic value. Some assessment tools attempt to reduce burden by keeping the symptom list short and allowing other patient-specific symptoms to be added at the end. However, it is still likely that symptoms not included in a symptom list will be missed, since patients are less likely to report symptoms not directly asked about [67]. In a study by White et al. (2009), only a third of symptoms were spontaneously self-reported by patients and the other two-thirds were only reported when systematically assessed [19]. The probability of not detecting common symptoms when not asked for, has been reported to be particularly high for some symptoms, such as dry mouth [68], and the intensity was then most often moderate to severe when first identified. Patients may not report mild symptoms because they are unaware of possible treatment [28], or because they do not perceive the problems to be important enough to mention since nurses and physicians do not ask about them [19, 30]. It has been noted that when dry mouth is reported as a mild symptom, it may not necessarily cause significant distress. However, when reported with moderate intensity, patients tend to consider it distressing [21]. A dry mouth can be linked to various conditions, including tooth decay, infections, speech difficulties, and challenges with chewing and swallowing [51, 69]. This is why early identification and appropriate measures may be potentially crucial to prevent development of these conditions.

### Strength and limitations

The main limitation of this study was the retrospective design comparing samples using two different ESAS versions in two different years rather than during the same year. In addition, ESAS-m was not validated to the same extent as ESAS-r, and their different items and wording limit the degree to which they can be directly compared. Both ESAS versions provided estimates of symptom prevalence and severity but did not assess the symptoms’ importance to the patient or the level of distress they caused. Thus, it is possible that the symptoms not asked about were not reported simply because the patient did not consider them to be important or distressing.

Although data were collected from patients in two different years, the demographic and medical data did not reveal statistical differences between the two groups. Additionally, seven symptoms were assessed similarly in both versions, and only the prevalence of tiredness differed between the versions, suggesting that the symptom patterns were similar for the patient groups assessed in both years. Missing information in the medical records on previous cancer treatment and functional status prevented us from analyzing associations between patient characteristics and symptom prevalence and severity. Finally, statistical power was low for analyses of the severity of “Other problems” due to the small numbers of patients reporting them. Nonetheless, our study showed the significant impact that the assessment method and tool can have on symptom reporting in a very vulnerable group of patients at the end of life.

## Conclusion

This retrospective study identified highly inconsistent reporting rates of the symptom dry mouth in palliative care due to differences in how the symptoms were assessed and supported our hypothesis that patients were less likely to report symptoms not directly asked about. Similar patterns were also evident for the symptoms of sleep problems and constipation, although constipation was listed as an alternative option within the question labeled “Other problem”. It is a methodological challenge to choose the right assessment tool for the right patient group due to complex individual needs and symptom burden. The limitations of commonly used and validated symptom instruments may lead to symptom under-reporting, interfere with effective symptom monitoring and delay or prevent treatment of both mild and severe symptoms.

### Electronic supplementary material

Below is the link to the electronic supplementary material.

Supplementary Material 1

## Data Availability

The dataset generated and analysed during the current study is stored on Lovisenberg Diaconal Hospital’s research server, in accordance with Norwegian ethical and legal requirements. Requests for access to an anonymized minimal data set can be sent to the corresponding author. Minimal anonymized data set can be shared, but only after approval from the Data Protection Officer at Lovisenberg Diaconal Hospital and the Regional Committees for Medical and Health Research.

## Abbreviations

ESAS: Edmonton Symptom Assessment System
ESAS-m: Edmonton Symptom Assessment System Norwegian modified version
ESAS-r: Edmonton Symptom Assessment System revised version
LDS: Lovisenberg Diaconal Hospital
LPCC: Lovisenberg Palliative Care Centre
NRS: Numeric Rating Scale
MCID: Minimal Clinically Important Difference
PC: Palliative Care
