# Supplementary material for: A comparison of the prevalence of dry mouth and other symptoms using two different versions of the Edmonton Symptom Assessment System on an inpatient palliative care unit
Source: BMC Palliat Care. 2024 Mar 16;23:75. doi: 10.1186/s12904-024-01405-7 (PMC10943902; doi:10.1186/s12904-024-01405-7)
Supplement: Supplementary file 1 — Supplementary Material 1 [file 12904_2024_1405_MOESM1_ESM.pdf]

Lovisenberg Diakonale Sykehus
Pionér i kompetanse og omsorg

PATIENTS ID

Name:..

Birth number:

How are you today?

ESAS (Edmonton Symptom Assessment System)

Date..............................

Time...............

Completed by: ☐ Patient ☐ Health Care Professional ☐ Patient with assistance

Pain at rest												
None	0	1	2	3	4	5	6	7	8	9	10	Worst Possible

Pain on movement												
None	0	1	2	3	4	5	6	7	8	9	10	Worst Possible

Tiredness												
None	0	1	2	3	4	5	6	7	8	9	10	Worst Possible

Nausea												
None	0	1	2	3	4	5	6	7	8	9	10	Worst Possible

Shortness of breath												
None	0	1	2	3	4	5	6	7	8	9	10	Worst Possible

Dry mouth												
None	0	1	2	3	4	5	6	7	8	9	10	Worst Possible

Lack of appetite												
None	0	1	2	3	4	5	6	7	8	9	10	Worst Possible

Constipation												
None	0	1	2	3	4	5	6	7	8	9	10	Worst Possible

Sleep problems												
None	0	1	2	3	4	5	6	7	8	9	10	Worst Possible

Anxious												
None	0	1	2	3	4	5	6	7	8	9	10	Worst Possible

Depressed												
None	0	1	2	3	4	5	6	7	8	9	10	Worst Possible

How do you feel overall today?												
Best wellbeing	0	1	2	3	4	5	6	7	8	9	10	Worst Possible
